# A description of the elevation of pericardial cortisol: cortisone ratio in patients with tuberculous pericarditis

**DOI:** 10.3389/fendo.2023.1127550

**Published:** 2023-05-25

**Authors:** Justin Tapiwa Shenje, Peter Raubenheimer, Lubbe Wiesner, Ian Ross

**Affiliations:** Department of Medicine, University of Cape Town, Cape Town, South Africa

**Keywords:** tuberculous pericarditis, cortisol, cortisone, cytokines, inflammation

## Abstract

Pulmonary tuberculosis is an inflammatory disease associated with an elevated cortisol/cortisone ratio at the site of infection and an array of cytokine changes. Tuberculous pericarditis is a less common but more lethal form of tuberculosis and has a similar inflammatory process in the pericardium. As the pericardium is largely inaccessible, the effect of tuberculous pericarditis on pericardial glucocorticoids is largely unknown. We wished to describe pericardial cortisolcortisone ratio in relation to plasma and saliva cortisol/cortisone ratios and the associated changes in cytokine concentrations. The median (interquartile range) of plasma, pericardial, and saliva cortisol concentration was 443 (379–532), 303 (257–384), and 20 (10–32) nmol/L, respectively, whereas the median (interquartile range) of plasma, pericardial, and saliva cortisone concentrations was 49 (35–57), 15.0 (0.0–21.7), and 37 (25–55) nmol/L, respectively. The cortisol/cortisone ratio was highest in pericardium with median (interquartile range) of 20 (13–445), followed by plasma of 9.1 (7.4–12.1) and saliva of 0.4 (0.3–0.8). The elevated cortisol/cortisone ratio was associated with elevated pericardial, interferon gamma, tumor necrosis factor–alpha, interleukin-6, interleukin-8, and induced protein 10. Administration of a single dose of 120 mg of prednisolone was associated with the suppression of pericardial cortisol and cortisone within 24 h of administration. The cortisol/cortisone ratio was highest at the site of infection, in this case, the pericardium. The elevated ratio was associated with a differential cytokine response. The observed pericardial cortisol suppression suggests that 120 mg of prednisolone was sufficient to evoke an immunomodulatory effect in the pericardium.

## 1 Introduction

Cortisol is the major endogenous glucocorticoid hormone and is fundamental to the stress response (1). Central to this function are its anti-inflammatory effects that reduce the potentially self-destructive effects of the host’s immune response during an infection (2). The suprachiasmatic nucleus of the hypothalamus regulates cortisol concentrations *via* the hypothalamic–pituitary–adrenal (HPA) axis, through its action on controlling the circadian rhythm and being sensitive to negative feedback inhibition (3). However, during acute stress or illness, plasma cortisol concentrations rise, and there is a loss of the diurnal variation (4). The elevated cortisol and disruption of the diurnal variation are thought to be due to the stimulation of the HPA axis, *via* the nerve fibers, lymphoid tissue, and the sympathetic medullary axis (5). Pro-inflammatory cytokines, for example, interleukin-1 (IL-1), also increase the production of corticotropic release hormone (5). Cortisol has anti-inflammatory effects, which it exerts by binding to the glucocorticoid receptor and by suppressing the aforementioned receptor function and repress production of IL-1β, IL-6, IL-8, IP-10, IL-12, and inducible nitric oxide synthase by neutrophiles and macrophages (5, 6).

Studies on tuberculosis (TB) have rarely examined the relationship between cortisol and cortisone at the site of infection. Baker et al., for example, have demonstrated the elevated cortisol/cortisone ratios in patients with pulmonary TB, yet this ratio is decreased with recovery (7). Moreover, with active PTB, the cortisol/cortisone ratio is higher in bronchial lavage fluid, compared with that in plasma. This is supportive of a shift in the cortisol-cortisone shuttle activity in favor of the production of cortisol at the site of infection (7, 8).

TB pericarditis (TBP) is a chronic granulomatous inflammatory disease caused by infection of the pericardium by *M. tuberculosis* (7). The immunological response to TBP leads to an accumulation of inflammatory exudate in pericardial space (9), followed by constrictive pericarditis in the short and long terms, respectively (10). About 8% of patients with TBP develop constrictive pericarditis, a condition that causes pericardial fibrosis, leading to stiffening of the pericardium, and compromises cardiac function (11). Immunomodulatory therapy with adjunctive corticosteroids is associated with a 40% reduction in constrictive pericarditis (12), emphasizing the importance of understanding the relationship between glucocorticoids and the immune system. As the site of infection in TBP is the pericardium and its inflammatory milieu (9), sampling the contents of the pericardial space should serve as a direct substrate from which the interaction between glucocorticoids and its respective inflammatory response is evaluated. In addition, obtaining samples from this anatomical locale is impossible, except under unusual circumstances, for example, TBP, where there is a substantial volume of fluid surrounding the heart, as routine sampling of this space in the absence of fluid will invariably cause cardiac injury (13). A systematic study of pericardial glucocorticoids and cytokine responses in TBP has never previously been performed, and studies evaluating this interaction in TBP will be pivotal in shaping our understanding of the changes that occur during this infective process, where, otherwise, it would have relied upon indirect evidence.

We previously published data on cytokine concentrations in the pericardium, plasma, and saliva of patients with TBP, in which we found that adjunctive corticosteroid therapy reduced saliva IL-1β, plasma IL-6, and pericardial IL-8 concentrations (14). As changes in cytokine concentrations are expected to occur in inflammatory and infective conditions (7, 8), these concentrations may be altered in response to an infected milieu, or it may be the result of changes in the cortisol/cortisone ratio at the site on infection, dampening the inflammatory response (7).

We hypothesized that there is a shift in the cortisol-cortisone shuttle enzyme activity, resulting in escalated cortisol concentrations, leading to a rise in the cortisol/cortisone ratio in patients infected with TBP, particularly of the pericardium. We further hypothesize that the rise in the cortisol/cortisone ratio is potentiated by a rise in pro-inflammatory cytokines at the site of infection, leading to the suppression of cortisone and relatively greater concentrations of cortisol.

Our objectives were, therefore, to describe the pericardial cortisol/cortisone ratio, a site that has never been previously evaluated for excursions of cortisol and cortisone, given its relative inaccessibility in patients with TBP, and to compare it to the more accessible plasma and saliva compartments, respectively, and to evaluate whether this rise corresponds to a rise in pericardial pro-inflammatory cytokines in the pericardium.

## 2 Materials and methods

**Figure 1:**
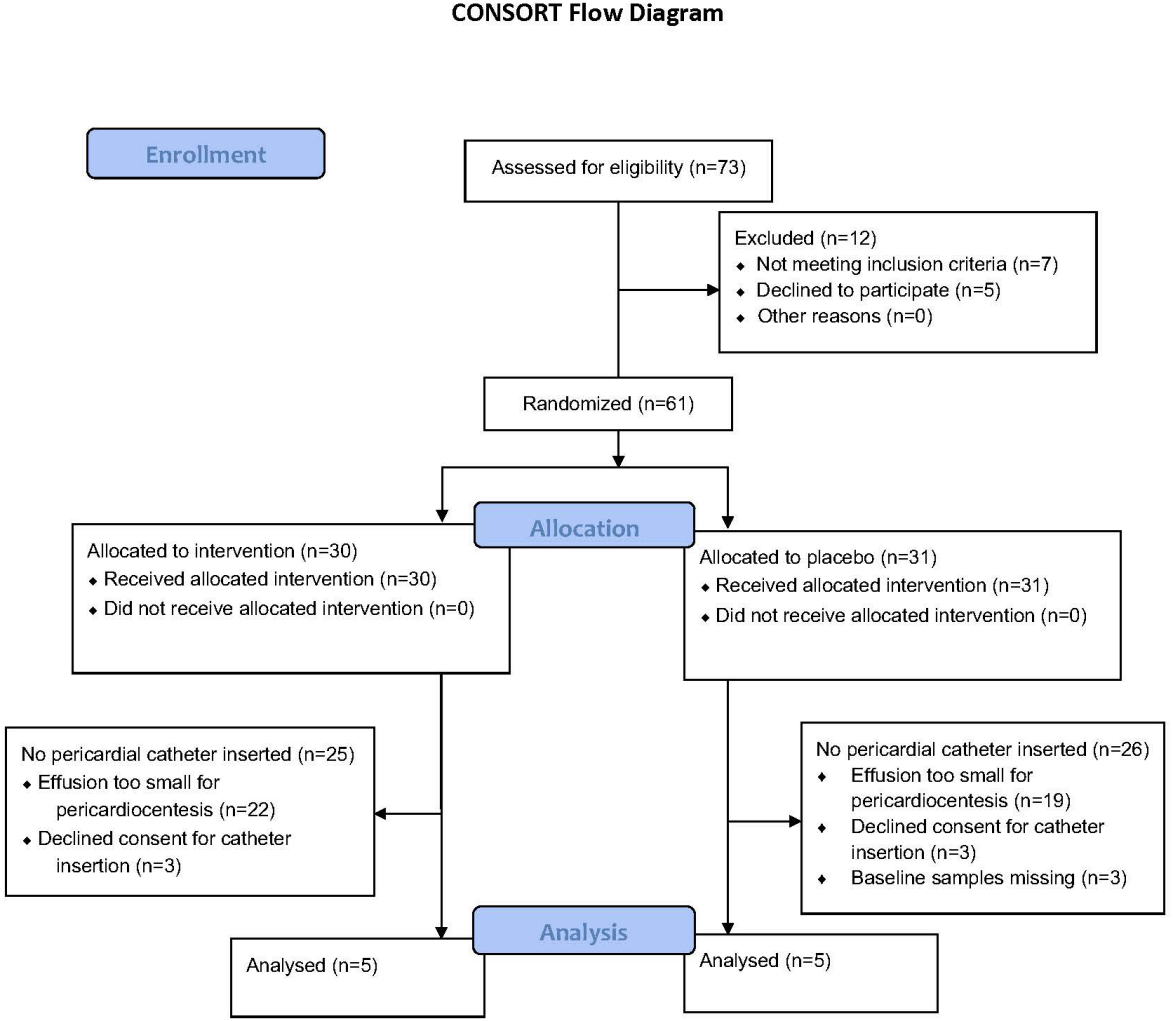
The number of participants screened and enrolled in each arm and the number of participants excluded from the analysis and the reason for exclusion.

The study was approved by the University of Cape Town Human Research Ethics Committee and adhered to the Declaration of Helsinki 2013 version 7.0 (15). Patients with TBP participated in a randomized control trial consisting of standard TB therapy with prednisolone or matched placebo: the Management of Tuberculous Pericarditis (IMPI) trial, Clinical Trials Registry Number: NCT00810849 (12). Only a small subset of the participants of the aforementioned trial was included in this sub-study if they presented to Groote Schuur Hospital in Cape Town, South Africa (a single site), from October 2012 to August 2014 (inclusive) and had a pericardial effusion amenable to pericardiocentesis. **Figure 1** shows the number of participants who were screened, enrolled, and allocated prednisolone or matching placebo in the sub-study. Participants who agreed to participate in the IMPI trial were also invited to take part in this sub-study, in which a pigtail pericardial drain was left *in situ* for 24 h to sample pericardial fluid continuously, and their endogenous glucocorticoids and prednisolone concentrations were assessed serially over a 24-h period. Patients were eligible for inclusion here if they were 18 years of age or older; had a pericardial effusion confirmed by echocardiography; had evidence of definite or probable TBP, according to the Tygerberg diagnostic score; and had less than 1 week of anti-tuberculous therapy (16, 17). Participants were commenced on World Health Organization (WHO)–recommended first-line anti-TB chemotherapy, consisting of rifampicin, isoniazid, ethambutol, and pyrazinamide, and randomized at a ratio of 1:1 to receive either adjunctive 120 mg of prednisolone or a matching placebo.

The active dose of prednisolone was administered orally as a single dose, comprising six tablets, each containing 20 mg of prednisolone, produced by Cadila Pharmaceuticals Ahmedabad, India, which also manufactured matching (in appearance and number) inactive placebo comprising sugar and starch. Either prednisolone or placebo was administered immediately after the baseline samples were taken for saliva, plasma, and pericardial fluid and subsequently administered after a period of 24 h and then daily for 28 days.

### 2.1 Sampling

Pericardial fluid, plasma, and saliva samples were then collected at baseline, before the administration of prednisolone, and then at the following time intervals after the administration of prednisolone or placebo: 0.5, 1, 2, 3, 5, 8, and 24 h, following insertion of the pigtail catheter. In view of the serial nature of the samples, a decision was made to collect single samples at each time point, rather than duplicate. Baseline venous blood, saliva, and pericardial fluid samples were collected simultaneously at bedside, following pericardiocentesis (as described above). Venous blood was extracted from a peripheral vein. Saliva was collected using Sarstedt salivettes (Nümbrecht, Germany). The swab remained inside the mouth for a period of 2 to 3 min while gentle chewing it and then was replaced in the salivette tube. Blood and pericardial fluid were collected in ethylenediamine tetraacetic acid (EDTA) tubes, each with a minimum of 4 ml, which were subsequently centrifuged at 2,000*g* for 10 min, and 1 ml of supernatant was pipetted into each cryotube and performed in duplicate. The cryotubes containing pericardial fluid and plasma were immediately stored at −80°CC in dedicated trays for subsequent analysis in a single batch. The salivettes were also stored at −80°CC to preserve stability, also using trays for vertical storage. On the day of analysis, they were thawed and centrifuged at 1,500*g* for 15 min. Time of day was recorded for each participant but differed in each case as patients presented at different times to the hospital, as were extremely unwell in each case. The time intervals were logged for subsequent sampling and administration of prednisolone or placebo. The continuous monitoring of the freezer revealed no accidental temperature fluctuations.

### 2.2 Endogenous glucocorticoid and pharmacological glucocorticoid analyses

Liquid chromatography–tandem mass spectrometry (LCMS/MS) was used to measure cortisol and cortisone concentrations in all our serial samples. Cortisol, cortisone, prednisolone, and prednisone concentrations were also determined by LCMS/MS assay, developed in the Division of Clinical Pharmacology, University of Cape Town, using the methodology described by the Food and Drug Administration (FDA) guidance for industry for bioanalytical method validation (18, 19). The assay was initially validated in plasma and then cross-validated in pericardial fluid and saliva. Charcoal stripped plasma, pericardial fluid, and saliva were used as controls. The limit of detection of assays was 0.5833 nmol/L; for concentrations that were measured below detection limits, a value of half the detection limit was assigned to allow calculation of relative ratios.

The assays were validated according to the FDA guidelines (19). Samples were processed with a liquid-liquid extraction method using ethyl acetate, followed by high-performance LCMS/MS detection using an AB SCIEX API 4000 instrument. An Agilent Zorbax-SB Phenyl Rapid Resolution HT 1.8 µm, 2.1 × 100 mm analytical column was used. Cortisol, cortisone, prednisolone, and prednisone were monitored at mass transitions of the protonated precursor ions at 363.2, 361.2, 361.2, and 359.2 to the product ions at 121.2, 163.1, 147.2, and 147.1, respectively. Cortisol-d4, cortisone-d8, prednisolone-d8, and prednisone-d4 stable isotope–labeled internal standards were used and monitored at mass transitions of the protonated precursor ions at 367.3, 369.3, 369.3, and 363.2 to the product ions 121.2, 168.2, 150.2, and 317.3, respectively. The calibration curves fitted quadratic (weighted by 1/concentration (2)) regressions over the ranges from 5.38 to 2758.6 nmol/L in plasma, pericardial fluid, and saliva.

### 2.3 Measurement of cytokine concentrations

Milliplex™kits (HCYTOMAG-60 K, Millipore, St. Charles, MO, USA) and the Bioplex200 reader from Bio-Rad were used to measure the following cytokines: IFN-γ, IL-1α, IL-1β, IL-6, IL-10, IL-12p40, TNF-α, IL-8, and IP-10, in pericardium, plasma, and saliva. When performing the cytokine analyses, a minimum volume of 250 μl. Samples were batched and analyzed on a single occasion. We did not perform our own validation study for pericardial fluid and saliva but employed the same methodology outlined by the manufacturer for the plasma cytokines that we analyzed (20). The details of the clinical validated reference ranges and limits of quantification and precision are outlined in the **supplementary appendix**.

### 2.4 Statistical analysis

Descriptive statistics were used to summarize patient data, namely, frequency and percentage for categorical data and median with interquartile range (IQR) for continuous data. The differences between participant allocated to placebo and those allocated to prednisolone were compared using the Mann–Whitney U-test and were performed using GraphPad Prism version 9.2.0 (San Diego, CA) and StataCorp version 17.0 (College Station, TX). Comparisons among the three sampled compartments (plasma, pericardial fluid, and saliva) were performed using the Friedman test, with *post-hoc* analysis between individual groups using the Wilcoxon test and were performed using SPSS version 28.0 (Chicago, IL). We reported *p*-values of less than 5% to represent significance.

## 3 Results

The participants screened and subsequently enrolled into this sub-study are shown in **Figure 1**. Ten participants were enrolled, five of whom were men. Six participants were HIV positive, and only one participant was on anti-retroviral therapy at the time of enrollment. Four of the six (67%) HIV-positive participants had CD4 counts below 200 cell/mm (3). Five participants had a diagnosis of confirmed TBP (50%), whereas the remaining participants had a diagnosis of probable TBP. All participants were of black African ancestry, except one who was of mixed ancestry. As shown in **Table 1**, there were no differences between participants allocated to placebo versus those allocated to prednisolone.

**Table 1:** Baseline characteristics of patients with TBP, allocated to either prednisolone or placebo, in addition to standard anti-TB therapy.

Variable	Total (n = 10)	Placebo (n = 5)	Treatment (n = 5)	p-value, placebo vs. treatment
Age (years)	38 (25–52)	45 (25–51)	31 (28–44)	0.7540
Sex, proportion of males	5 (50%)	2 (40%)	3 (60%)	0.5485
Weight (kg)	66 (53–72)	70 (53–73)	66 (52–72)	0.5970
HIV positive	6 (60%)	3 (60%)	3 (60%)	1.0
On HAART	1 (10%)	1 (20%)	0 (0%)	0.3173
CD4 count (cells/mm (3)	319 (139–485)	403 (240–603)	139 (135–319)	0.2207
Creatinine (μmol/L)	82 (65–105)	115 (95–189)	65 (45–82)	0.0635
Serum globulin (g/L)	55 (38–56)	52 (39–56)	55 (38–56)	1.000
Pericardial protein (g/L)	66 (62–68)	65 (56–72)	66 (62–68)	0.9021
Pericardial Adenine deaminase (IU/L)	53 (33–92)	72 (42–113)	51 (33–57)	0.4724
Definitive TBP	5 (50%)	2 (40%)	3 (60%)	0.5485

Categorical data represented in n (%), and continuous data represented in median (interquartile range).

### 3.1 Cortisol measurements

**Figure 2:**
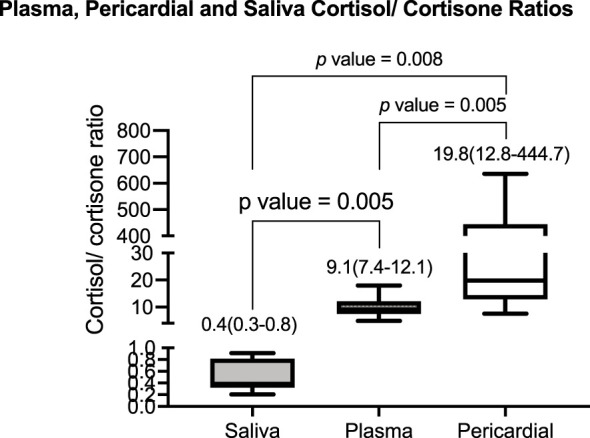
The baseline median (interquartile range) of the cortisol/cortisone ratio in saliva, plasma, and pericardial fluid was 0.4 (0.3–0.8), 9.1 (7.4–12.1), and 19.8 (12.8–444.7), respectively. The p-value indicates the pairwise comparison for the post-hoc Wilcoxon test.

At baseline, the median (IQR) cortisol concentration in the pericardium was 303 (258–385) nmol/L, plasma cortisol was 443 (379–523) nmol/L, and salivary cortisol was 20 (10–32) nmol/L. The median (IQR) cortisone concentration was 15.0 (0.6–21.7) nmol/L, 49 (35–58) nmol/L, and 37 (25–55) nmol/L, in pericardium, plasma, and saliva, respectively. The relevant median (IQR) cortisol/cortisone ratios in the three compartments were significantly different from each other at 20 (13–445) in the pericardium, 9.1 (7.4–12.1) in plasma, and 0.4 (0.3–0.8) in saliva (**Figure 2**). See **supplementary appendix**.

### 3.2 Cytokine measurements

#### 3.2.1 Comparison of cytokines in plasma in patients with TBP versus reference range derived from plasma of healthy controls

The cytokine concentrations in the three compartments, pericardium, plasma, and saliva are shown in **Table 2**. The plasma cytokine concentrations were compared with their respective reference ranges, as determined in healthy individuals’ plasma, as shown in **Table 2**. The plasma cytokine concentrations of IL-1α, IL-1β, IL-8, IL-10, IP-10, and IL-12P40 were comparable with their reference ranges (21–24), whereas TNF-α (2-fold), IL-6 (10-fold), IL-8 was (5-fold), and IFN- (200-fold) are greater than their reference ranges (20). See **supplementary appendix**.

**Table 2:** Plasma, pericardial, and saliva cytokine concentrations and their respective plasma reference ranges.

Cytokine	Plasma reference range, pg/ml, median (10th–90th %tile) (20)	Plasma median (IQR), pg/ml	Pericardial median (IQR), pg/ml	Saliva median (IQR), pg/ml	*p*-value, Friedman test
IFN-γ	0.1 (0.1–0.3)	28 (11–163)	2,061 (690–2,765)	18 (10–23)	**0.0011**
TNF-α	14 (11–19)	33 (22–58)	197 (92–430)	16 (10–80)	**0.0043**
IL-1α	0 (0–0)	0 (0–0)	0 (0–62)	2,999 (979–9,910)	**0.0002**
IL-1β	0 (0–0)	0 (0–0)	8 (0–114)	31 (21–180)	**0.0039**
IL-6	3.4 (1.3–10)	23 (18–75)	8,816 (8,225–9,953)	11 (5–32)	**0.0009**
IL-8	4.4 (2.4–6.5)	23 (14–31)	8,840 (1,841–11,635)	401 (93–1,256)	**0.0001**
IL-10	4.8 (3.7–8.8)	4 (0–9)	34 (23–77)	17 (2–36)	0.0054
IP-10	64 (33–103)	82 (33–155)	1,414 (1,002–1,574)	1,061 (201–2,420)	**0.0031**
IL-12P40	0 (0–0)	0 (0–0)	0 (0–1)	24 (0–48)	**0.0037**

Source of recommended plasma reference ranges for the following cytokines (20). The Friedman test was used to evaluate whether there is a difference between cytokine concentrations in all three compartments.

The bold p values indicate that the p value was less than 0.05 and were thus clinically significant.

#### 3.2.2 Comparison of pericardial cytokines from patients with TBP with plasma cytokine of healthy controls

Because of the absence of normal reference ranges for pericardial cytokine concentrations, the pericardial cytokine concentrations were compared with their respective plasma reference ranges, as determined in healthy individuals’ plasma, which are also shown in **Table 2**. Pericardial IL1α, IL-1β, and IL-12P40 were similar to their respective plasma reference ranges, whereas pericardial cytokine INF-γ (log_3_), TNF-α (10-fold), IL-6 (log_3_), IL-8 (log_3_), IL-10 (7-fold), and IP-10 (20-fold) are higher than their respective plasma reference ranges (21–24). Saliva IFN (log-2), IL-1α (log_3_), IL-1β (30-fold), IL-6 (log_3_), IL-8 (log_3_), IL-10 (3-fold), IP-10 (20-fold), and IL-12P40 (20-fold) are lower than their reference ranges, respectively, whereas TNF-α was comparable to its respective reference range (20). See **supplementary appendix**.

#### 3.2.3 Comparison of pericardial, saliva, and plasma cytokines in patients with TBP

For the different compartments, there were marked differences in cytokine concentrations. For example, pericardial IL-6 was 600-fold greater than saliva and plasma concentrations. In addition, INF-γ was about 100-fold greater in the pericardium, compared with saliva and plasma concentrations; however, the latter two were similar. TNF-α was greatest in pericardium and was eight-fold greater than that of saliva and plasma concentrations, respectively. Saliva IL-1α, by contrast, was elevated by a magnitude of log_3_, compared with plasma and pericardial concentrations, which were similar. IL-1β was elevated in saliva, lower in pericardium, and least in plasma. The relative proportion of IL-8 concentrations in plasma, pericardium, and saliva was 1:20:400. The concentrations of IL-10 were similar in pericardium and saliva, but IL-10 was notably lower in plasma. IP-10 was similar in pericardium and saliva but was 15-fold lower in plasma. Saliva IL-12P40 was considerably higher, compared with plasma and pericardium, both of which were virtually undetectable.

### 3.3 Prednisolone and cortisol/cortisone ratios

The cortisol/cortisone ratio was highest in pericardial fluid median (IQR) 20 (13–445), followed by plasma median (IQR) of 9.1 (7.4–12) and then lowest in saliva median (IQR) of 0.4 (0.3–0.8). In the placebo arm, the cortisol and cortisone concentrations were unchanged throughout the 24-h period in all three matrices, suggesting a high degree of reproducibility. In participants allocated to prednisolone, there was a rapid (from as early as 3 h) and sustained reduction of cortisol and cortisone concentration for all three compartments.

**Figure 3:**
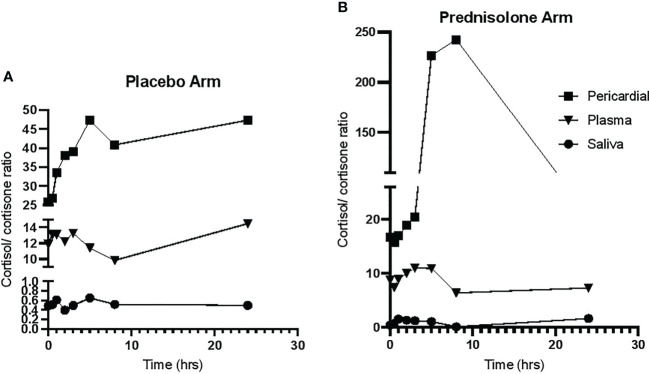
The cortisol/cortisone ratio over a 24-h period. **(A)** The cortisol/cortisone ratio in participants allocate to placebo. **(B)** The ratio in participants allocated to prednisolone.

The cortisol/cortisone ratios in the placebo arm did not change significantly in all the matrices over the 24-h period, as shown in **Figure 3A**. **Figure 3B** shows that, after a period of 5 h in patients who received prednisolone, the pericardial cortisol/cortisone ratio dramatically increase followed by a decrease after 8 h, whereas these ratios in plasma and saliva were unchanged over the 24 h.

## 4 Discussion

This is the first examination of pericardial cortisol and cortisone levels and their relationship to cytokine concentrations in patients with TBP. We found that the pericardium has a high concentration of cortisol, close to that of the plasma compartment and, by contrast, much higher than in the salivary compartment, but a lower cortisone concentration. This resulted in an elevated pericardial cortisol/cortisone ratio when compared with plasma and saliva. In addition, most key cytokines measured were markedly elevated in the pericardium versus the plasma.

Local cortisol-to-cortisone equilibriums are mostly dependent on the activity of local expression of the enzyme 11HSD-1 and 11HSD-2. In salivary glands, for example, there is a well-established high expression of 11HSD-2, resulting in local conversion of cortisol to cortisone and a low cortisol/cortisone ratio (25). In view of the absence of direct evidence in the setting of TBP, we speculate that there is an increased expression of 11HSD-1 in the endothelial cells of the pericardial vasculature where it is expressed (7, 8), but this has not yet been proven. Furthermore, our hypothesis is that this is driven by the increased local concentrations of pro-inflammatory cytokines such as TNF-α and IL-1β. Moreover, our conjecture is that the aforementioned proinflammatory cytokines result in a shift in favor of pericardial cortisol rather than cortisone, compared with plasma (26, 27).

These findings are consistent with the findings of Baker et al., who showed an elevated cortisol/cortisone ratio in bronchoalveolar lavage fluid in patients with pulmonary TB despite a normal central control of glucocorticoid production, in keeping with altered peripheral metabolism at the site of infection (7).

We do not know whether the altered local glucocorticoid equilibrium is potentially beneficial, harmful, or not of pathogenic relevance at all. TB is an inflammatory disease, and much of the damage in PTB is due to an inflammatory response (9). The elevated cortisol/cortisone ratio may be of benefit in patients with TBP, through paring down the inflammatory response. Adjunctive corticosteroid therapy has been prescribed for the treatment of TBP patients, with the view to curtailing the inflammatory resonse (11). Baker et al., however, speculated that increased active glucocorticoids in the lungs of patients with pulmonary TB could be harmful; it could, for example, explain the increased production of TGF-β and IL-10 in human TB and the impaired macrophage function. Either way, manipulation of the cortisol/cortisone equilibrium with, for example, 11HSD-1 inhibitors could be beneficial or harmful in patients with TBP and would need further investigation.

Previous work has shown that providing supraphysiological doses of prednisolone in a clinical study of TBP has conferred clinical benefit in HIV-negative patients, by reducing duration of hospitalisation and incidence of constrictive pericarditis, a lethal complication (12). Our study was not designed to determine the clinical benefit but to determine its action on suppression of endogenous glucocorticoids in all compartments. Administering supraphysiologic prednisolone led to cortisol and cortisone suppression in all three compartments, plasma pericardium, and saliva. Plasma and saliva cortisol and cortisone suppression in response to administration of supra-physiological glucocorticoids has been well documented (28, 29); however, this is the first time that it has been demonstrated in the pericardium. Administration of prednisolone caused the pericardial cortisol/cortisone ratio to initially increase then decline; this is despite a rapid and sustained decline in pericardial cortisol. The median pericardial cortisone concentrations from 5 h onward dropped to below 1 nmol/L; therefore, according to L’Hospital’s Rule (30), a ratio approaches infinity as the function of the denominator approaches zero, and, thus, the rise in pericardial cortisol/cortisone ratio may be artifactually high due to the extremely low pericardial cortisone concentrations. The later decline in cortisol/cortisone ratio is due to the extremely low cortisol concentrations at later timepoints. Overall, our data support that glucocorticoid therapy using prednisolone suppressed pericardial cortisol and cortisone concentrations in a similar manner to plasma and saliva cortisol and cortisone concentrations. In addition, the subsequent follow-up data during the 24-h period for those individuals who received prednisolone corroborates the reliability of the data in **Figure 3**.

It is apparent that the vast majority of our participants were of black African ancestry. Black and white women did not differ in the study evaluating the HPA axis using the 24-h urinary excretion of cortisol and dexamethasone suppressibility matched for age, weight, body mass index, and body surface area (31). Despite the majority of our participants being of black African ancestry, it is likely that these data are generalizable to other ethnic population groups.

However, the study had a number of weaknesses, it is composed of a small sample size, and sampling was limited to the first 24-h time period. It is likely that additional physiological responses occurred beyond this period, for example, during recovery. This study did not undertake cortisol-cortisone shuttle enzyme expression studies, thus relying on indirect evidence that it conferred an immune modulatory response, which warrants confirmation in the future. The participants were mainly of black African ancestry; therefore, there is uncertainty whether these findings are generalizable to other populations. We did not perform our own validation study on the cytokines but used the prescribed reference values. This represents an additional weakness of our study. Moreover, the reference ranges in plasma may not be applicable to pericardial fluid, thus invoking another degree of uncertainty in the interpretation of these data.

## 5 Conclusion

This pilot study was able to show a greater inflammatory response in the pericardium of patients with TBP, as evidenced by the following elevated proinflammatory cytokines: INF-γ, TNF-α, IL-6, and IL-8. In the pericardium, there was also a raised cortisol/cortisone ratio, compared with plasma and saliva. In light of the previously documented evidence for the beneficial effect of adjunctive corticosteroids in patients with TBP, the elevated endogenous pericardial cortisol/cortisone ratio may have a partial immunomodulatory and, potentially, an immunostimulatory effect. 

## Data availability statement

The original contributions presented in the study are included in the article/**Supplementary Material**. Further inquiries can be directed to the corresponding author.

## Ethics statement

The studies involving human participants were reviewed and approved by University of Cape Town Human Research Ethics Committee. The patients/participants provided their written informed consent to participate in this study.

## Author contributions

JS, PR, and IR contributed to conception and design of the study. JS organized the database. JS and PR performed the statistical analysis. JS wrote the first draft of the manuscript, whereas PR and IR contributed to manuscript revision. All authors contributed to the article and approved the submitted version.
