# Supplementary material for: A description of the elevation of pericardial cortisol: cortisone ratio in patients with tuberculous pericarditis
Source: Front Endocrinol (Lausanne). 2023 May 25;14:1127550. doi: 10.3389/fendo.2023.1127550 (PMC10248178; doi:10.3389/fendo.2023.1127550)
Supplement: Supplementary file 1 [file DataSheet_1.pdf]

Supplementary Appendix

# A description of the elevation of pericardial cortisol: cortisone ratio in patients with tuberculous pericarditis

**Justin Shenje[1*], Peter Raubenheimer[1], Lubbe Wiesner, Ian Ross[1]**

## 1. Participant characteristics

Table describing the baseline characteristics of the participants who were enrolled in the study.

*Table 1: Participant Characteristics*

| PID | Age | wt | Sex | HIV | HAART | CD4 | Creat | Globulin | Pred | P protein | ADA | Culture |
|---|---|---|---|---|---|---|---|---|---|---|---|---|
| **PID1** | 24 | 66 | female | + | no | 42 | 43 | 56 | Yes | 68 | 57,1 | + |
| **PID2** | 56 | 82 | male | - | N/A | 485 | 121 | 30 | No | 62 | 133 | + |
| **PID3** | 31 | 72 | male | - | N/A | 319 | 82 | 36 | Yes | 55 | 119,4 | + |
| **PID4** | 51 | 53 | female | + | yes | 159 | 80 | 56 | No | 50 | 52,5 | - |
| **PID5** | 24 | 45 | female | + | no | 321 | 257 | 56 | No | 67 | 92,1 | + |
| **PID6** | 27 | 66 | female | + | no | 135 | 45 | 55 | Yes | 70 | 25,9 | + |
| **PID7** | 44 | 52 | female | + | no | 139 | 65 | 56 | Yes | 66 | 51 | - |
| **PID8** | 59 | 66 | male | - | N/A | 874 | 97 | 38 | Yes | 62 | 33,2 | - |
| **PID9** | 45 | 70 | male | - | N/A | 721 | 109 | 47 | No | 76 | 32,4 | - |
| **PID10** | 25 | 73 | male | + | no | N/A | 73 | | No | 55 | 38 | - |

**Key for Table 1:**

| | |
|---|---|
| PID | Participant identifier. |
| Age | Age in years. |
| Wt | Weight in kilograms |
| HIV | Human immunodeficiency virus, + for HIV-positive and – for HIV-negative |
| HAART | Highly active anti-retroviral therapy |
| Creat | Serum creatinine |
| Globulin | Serum globulin, calculated by subtracting serum albumin from total serum protein. |
| Pred | Whether participant was allocated to prednisolone. |
| P protein | Total pericardial protein |
| ADA | Adenine deaminase |
| Culture | Sputum M. tuberculosis culture using Mycobacterial growth indicator tube (MGIT) |

## 2. Endogenous glucocorticoid methods and analyses

Cortisol, cortisone, prednisolone and prednisone concentrations were also determined by LCMS/MS assay, using the methodology described by Food and Drug Administration (FDA) guidance for industry for bioanalytical method validation.[1] Tables 2-8 give details of the assay, while Tables 9-14 gives the raw data in nmol/L with a lower limit of quantification of 0.5833 nmol/L.

**Accuracy and precision**

The accuracies of the assay were between 85% and 115% during validation and sample analysis for all analytes. The percentage coefficient of variation (precision) of the assay was less than 15% during validation and sample analysis for all analytes.

**Internal standards**

Stable isotope labelled internal standards were used: cortisol-d4, cortisone-d8, prednisolone-d8, and prednisone-d4.

**Extraction procedure**

Samples were thawed on ice and briefly vortex-mixed and 200 µL transferred to microcentrifuge tubes. Blank matrix containing the internal standards (500 ng/mL cortisol-d4 and cortisone-d8, and 2000 ng/mL prednisolone-d8 and prednisone-d4) were added to the samples. Ethyl acetate (1000 µL) was added and vortex-mixed for 30 seconds. The samples were centrifuged at 5750 G for 5 minutes and 500 µL of the supernatant was transferred to clean microcentrifuge tubes. The samples were dried at 37°C for 20 minutes and reconstituted with 200 µL of mobile phase and vortex-mixed for 30 seconds and transferred to 96-well plates. Ten microliters were injected onto the HPLC column.

**Chromatography**

Isocratic chromatography was performed on an Agilent Zorbax-SB Phenyl Rapid Resolution HT 1.8 µm, 2.1 x 100 mm analytical column. The mobile phase consisted of acetonitrile and 0.1% formic acid in water (35:65, v/v) and was delivered at a flow rate of 140 µL/min. The

column compartment temperature was set at 20°C and the autosampler temperature was set at 10°C.

*Table 2: Retention times*

| Analyte | Time (min) |
|---|---|
| Analyte: Cortisol | 6.4 |
| ISTD: Cortisol-d4 | 6.4 |
| Analyte: Cortisone | 7.8 |
| ISTD: Cortisone-d8 | 7.8 |
| Analyte: Prednisolone | 7.0 |
| ISTD: Prednisolone-d8 | 7.0 |
| Analyte: Prednisone | 7.9 |
| ISTD: Prednisone-d4 | 7.9 |

**Mass spectrometer**

A SCIEX API 4000 instrument was used in the electrospray ionization mode. Cortisol, cortisone, prednisolone and prednisone were monitored at mass transitions of the protonated precursor ions 363.2, 361.2, 361.2, and 359.2 to the product ions 121.2, 163.1, 147.2, and 147.1, respectively. Cortisol-d4, cortisone-d8, prednisolone-d8 and prednisone-d4 stable isotope labelled internal standards were used and monitored at mass transitions of the protonated precursor ions 367.3, 369.3, 369.3, and 363.2 to the product ions 121.2, 168.2, 150.2, and 317.3, respectively.

*Table 3: Electro Spray Ionization Settings*

| | |
|---|---|
| **Nebuliser gas (Gas 1) (arbitrary unit)** | 50 |
| **Turbo gas (Gas 2) (arbitrary unit)** | 60 |
| **CUR (curtain gas) (arbitrary unit)** | 30 |
| **CAD (collision gas) (arbitrary unit)** | 6 |
| **TEM (Source Temperature) (°C)** | 300 |
| **IS (Ion Spray Voltage) (V)** | 5500 |

*Table 4: MS/MS Settings Cortisol*

| | Cortisol | Cortisol-d4 |
|---|---|---|
| **Protonated molecular ion mass (m/z) $[M+H]^+$** | 363.2 | 367.3 |
| **Product ion mass (m/z) Quantifier** | 121.2 | 121.2 |
| **Product ion mass (m/z) Qualifier** | 91.2 | 97.3 |
| **Dwell time (ms)** | 150 | 150 |
| **Declustering potential (V)** | 66 | 71 |
| **Entrance potential (V)** | 10 | 10 |
| **Collision energy (eV)** | 33 | 33 |
| **Collision cell exit potential (V)** | 10 | 10 |

*Table 5: MS/MS Settings Cortisone*

| | Cortisone | Cortisone-d8 |
|---|---|---|
| **Protonated molecular ion mass (m/z) $[M+H]^+$** | 361.2 | 369.3 |
| **Product ion mass (m/z)   Quantifier** | 163.1 | 168.2 |
| **Product ion mass (m/z)   Qualifier** | 121.1 | 105.1 |
| **Dwell time (ms)** | 150 | 150 |
| **Declustering potential (V)** | 76 | 86 |
| **Entrance potential (V)** | 10 | 10 |
| **Collision energy (eV)** | 33 | 35 |
| **Collision cell exit potential (V)** | 10 | 12 |

*Table 6: MS/MS Settings Prednisolone*

| | Prednisolone | Prednisolone-d8 |
|---|---|---|
| **Protonated molecular ion mass (m/z) $[M+H]^+$** | 361.2 | 369.3 |
| **Product ion mass (m/z)   Quantifier** | 147.2 | 150.2 |
| **Product ion mass (m/z)   Qualifier** | 171.1 | 151.2 |
| **Dwell time (ms)** | 150 | 150 |
| **Declustering potential (V)** | 41 | 46 |
| **Entrance potential (V)** | 10 | 10 |
| **Collision energy (eV)** | 31 | 31 |
| **Collision cell exit potential (V)** | 12 | 14 |

*Table 7: MS/MS Settings Prednisolone*

| | Prednisone | Prednisone-d4 |
|---|---|---|
| **Protonated molecular ion mass (m/z) $[M+H]^+$** | 359.2 | 363.2 |
| **Product ion mass (m/z)   Quantifier** | 147.1 | 317.3 |
| **Product ion mass (m/z)   Qualifier** | 171.1 | 269.1 |
| **Dwell time (ms)** | 150 | 150 |
| **Declustering potential (V)** | 56 | 51 |
| **Entrance potential (V)** | 10 | 10 |
| **Collision energy (eV)** | 37 | 19 |
| **Collision cell exit potential (V)** | 8 | 8 |

*Table 8: Scan Description*

| | |
|---|---|
| **Scan Type** | MRM |
| **Polarity** | Positive |
| **Pause Time (ms)** | 5 |

*Table 9: Pericardial Cortisol*

| Time | PID1 | PID2 | PID3 | PID4 | PID5 | PID6 | PID7 | PID8 | PID9 | PID10 |
|---|---|---|---|---|---|---|---|---|---|---|
| 0 | 216,8 | 361,4 | 205,5 | 317,2 | 270,9 | 244,4 | 303,4 | 438,6 | 275,9 | 540,7 |
| 0,5 | 187,6 | 369,7 | | 284,1 | 408,2 | 256,5 | 234,8 | 377,9 | 260,1 | 504,8 |
| 1 | 165,0 | 361,4 | 230,6 | 333,8 | 364,1 | 200,0 | 314,4 | 366,9 | 252,4 | 400,0 |
| 2 | 180,4 | 413,8 | 167,7 | 325,5 | 292,4 | 145,4 | 336,5 | 353,1 | 309,0 | 377,9 |
| 3 | 149,2 | 413,8 | 212,4 | 289,7 | 292,4 | 167,7 | 210,5 | 416,5 | 257,3 | 433,1 |
| 5 | 112,8 | 899,3 | 166,6 | 358,6 | 289,7 | 195,9 | 225,4 | 151,7 | 275,3 | 361,4 |
| 8 | 68,1 | 366,9 | 120,8 | 408,2 | 347,6 | | 128,3 | | 240,0 | |
| 24 | 29,0 | 265,1 | 51,3 | 336,5 | 303,4 | | 65,9 | 37,5 | 256,5 | |

*Table 10: Plasma Cortisol*

| Time | PID1 | PID2 | PID3 | PID4 | PID5 | PID6 | PID7 | PID8 | PID9 | PID10 |
|---|---|---|---|---|---|---|---|---|---|---|
| 0 | 339,3 | 422,1 | 549,0 | 477,2 | 582,1 | 463,4 | 311,7 | 526,9 | 419,3 | 391,7 |
| 0,5 | 121,9 | 554,5 | | 584,8 | 551,7 | 218,2 | 197,2 | 311,7 | 383,4 | 213,5 |
| 1 | 115,0 | 526,9 | 191,7 | 617,9 | 485,5 | 211,3 | 166,6 | 203,0 | 353,1 | 185,4 |
| 2 | 100,4 | 433,1 | 126,3 | 408,2 | 488,3 | 147,3 | 77,0 | 132,7 | 325,5 | 170,5 |
| 3 | 109,2 | 397,2 | 107,9 | 303,4 | 524,1 | 133,5 | 54,6 | 101,2 | 435,9 | 173,0 |
| 5 | 80,0 | 609,7 | 80,0 | 220,4 | 507,6 | 110,9 | 45,0 | 77,0 | 394,5 | 128,8 |
| 8 | 67,0 | 441,4 | 57,9 | 264,5 | 430,3 | | 33,7 | | 394,5 | |
| 24 | 48,0 | 411,0 | 177,9 | 623,4 | 427,6 | | 93,5 | 53,5 | 273,4 | |

*Table 11: Saliva Cortisol*

| Time | PID1 | PID2 | PID3 | PID4 | PID5 | PID6 | PID7 | PID8 | PID9 | PID10 |
|---|---|---|---|---|---|---|---|---|---|---|
| 0 | 8,3 | 18,8 | 32,3 | 12,7 | 45,0 | 9,7 | 17,5 | 5,4 | 21,9 | |
| 0,5 | 12,6 | 27,9 | | 19,1 | 33,1 | 22,7 | 65,4 | 15,7 | 10,5 | |
| 1 | 15,5 | 22,8 | 52,4 | 31,4 | 37,8 | 49,1 | 41,7 | | 13,0 | |
| 2 | 17,7 | 19,5 | 104,0 | 14,1 | 28,7 | 47,4 | 19,6 | 22,6 | 5,8 | |
| 3 | 27,4 | 16,0 | 24,2 | 9,5 | 32,3 | 36,4 | 12,7 | 14,4 | 14,7 | |
| 5 | 22,7 | 64,8 | 13,0 | 14,2 | 27,9 | 19,8 | 9,2 | | 18,0 | |
| 8 | 19,4 | 29,8 | BLQ | 8,2 | 21,1 | | BLQ | | 16,1 | |
| 24 | BLQ | 43,6 | 17,8 | 24,4 | 19,6 | | 128,8 | | 13,7 | |

*Table 12: Pericardial cortisone*

| Time | PID1 | PID2 | PID3 | PID4 | PID5 | PID6 | PID7 | PID8 | PID9 | PID10 |
|---|---|---|---|---|---|---|---|---|---|---|
| 0 | 15,0 | 23,0 | BLQ | BLQ | BLQ | 26,6 | 17,8 | 18,6 | 36,1 | 20,4 |
| 0,5 | 12,0 | 21,7 | | BLQ | 6,3 | 26,1 | 14,0 | 15,3 | 38,1 | 18,4 |
| 1 | 10,1 | 20,2 | BLQ | BLQ | 6,5 | 17,4 | 18,1 | 13,2 | 37,3 | 11,4 |
| 2 | 9,4 | 22,1 | BLQ | BLQ | BLQ | 7,5 | 17,3 | 11,1 | 43,9 | 9,5 |
| 3 | 7,2 | 21,2 | BLQ | BLQ | BLQ | 8,8 | 9,8 | 12,7 | 37,5 | 10,6 |
| 5 | BLQ | 37,0 | BLQ | BLQ | BLQ | 10,6 | 9,7 | BLQ | 38,1 | 7,1 |
| 8 | BLQ | 15,0 | BLQ | BLQ | 5,5 | | BLQ | | 33,1 | |
| 24 | BLQ | 6,1 | BLQ | 5,7 | BLQ | | 6,7 | BLQ | 43,9 | |

*Table 13: Plasma cortisone*

| Time | PID1 | PID2 | PID3 | PID4 | PID5 | PID6 | PID7 | PID8 | PID9 | PID10 |
|---|---|---|---|---|---|---|---|---|---|---|
| 0 | 35,3 | 32,2 | 62,3 | 39,8 | 32,0 | 53,9 | 47,3 | 55,6 | 85,3 | 50,3 |
| 0,5 | 15,7 | 27,5 | | 44,2 | 33,4 | 30,0 | 31,1 | 33,9 | 84,0 | 31,7 |
| 1 | 11,7 | 27,7 | 21,0 | 46,7 | 31,4 | 16,8 | 24,7 | 25,7 | 89,2 | 18,6 |
| 2 | 6,3 | 26,5 | 12,2 | 38,9 | 28,4 | 8,3 | 8,0 | 14,0 | 83,1 | 13,5 |
| 3 | 7,3 | 29,5 | 9,9 | 37,0 | 33,4 | 9,1 | 6,9 | 8,8 | 67,6 | 11,4 |
| 5 | 0,0 | 37,5 | 9,2 | 26,6 | 29,2 | 9,5 | 6,2 | 6,6 | 79,2 | 10,8 |
| 8 | 0,0 | 45,0 | 8,7 | 26,1 | 23,6 | | 6,0 | | 74,2 | |
| 24 | 6,5 | 37,5 | 22,8 | 33,9 | 20,9 | | 13,9 | 6,1 | 55,9 | |

*Table 14: Saliva cortisone*

| Time | PID1 | PID2 | PID3 | PID4 | PID5 | PID6 | PID7 | PID8 | PID9 | PID10 |
|---|---|---|---|---|---|---|---|---|---|---|
| 0 | 22,8 | 32,0 | 35,6 | 34,8 | 52,3 | 38,1 | 23,0 | 15,0 | 107,9 | |
| 0,5 | 25,7 | 43,9 | | 41,7 | 59,5 | 46,1 | 23,6 | 17,9 | 74,8 | |
| 1 | 20,3 | 37,8 | 36,1 | 52,0 | 57,0 | 21,3 | 27,0 | | 78,1 | |
| 2 | 15,8 | 26,1 | 21,4 | 41,1 | 66,2 | 20,5 | 15,2 | 36,4 | 37,5 | |
| 3 | 14,0 | 26,7 | 20,4 | 26,0 | 51,7 | 22,8 | 12,1 | 24,2 | 83,1 | |
| 5 | 14,7 | 60,0 | 15,7 | 21,0 | 45,3 | 20,9 | 8,2 | | 90,1 | |
| 8 | 14,4 | 40,3 | 9,3 | 24,7 | 30,9 | | 6,4 | | 90,4 | |
| 24 | 5,7 | 24,0 | 11,0 | 58,9 | 34,5 | | 20,2 | | 77,8 | |

## 3. Cytokine data

Plasma, pericardial and saliva cytokine concentrations are shown in Tables 8, 9 and 10. These cytokines were measured using Milliplex™kits (HCYTOMAG-60 K, Millipore, St Charles, MO, USA) on the Bioplex200 reader from Biorad. The minimum required sample volume was 250 μL. All cytokine concentrations were measured in pg/mL. Details on lower limit of quantification (LLOQ), upper limit of quantification (ULOQ) and precision of the instrument for each cytokine are shown below in Table 11:[2]

https://olink.com/content/uploads/2023/02/olink-flex-validation-data.pdf .

*Table 15: Pericardial fluid Cytokines*

| PID | IFN-G | IL-10 | IL-12P40 | IL1A | IL-1B | IL-6 | IL-8 | TNF-A | IP-10 |
|---|---|---|---|---|---|---|---|---|---|
| PID1 | 1585 | 28 | BLQ | BLQ | BLQ | 9937 | 3725 | 180 | 1528 |
| PID2 | 2536 | 37 | 6 | BLQ | 81 | 1980 | 11672 | 213 | 1662 |
| PID3 | 2725 | 63 | BLQ | 297 | 214 | 9277 | 11623 | 545 | 1545 |
| PID4 | 891 | 72 | BLQ | 11 | 40 | 8781 | 9329 | 66 | 1306 |
| PID5 | 2883 | 20 | BLQ | 210 | 502 | 8850 | 10038 | 665 | 2157 |
| PID6 | 85 | 31 | BLQ | BLQ | BLQ | 8532 | 1050 | 52 | 300 |
| PID7 | 980 | 23 | BLQ | BLQ | BLQ | 8533 | 8351 | 162 | 1392 |
| PID8 | 10000 | 21 | 5 | BLQ | 3 | 10000 | 1973 | 392 | 1435 |
| PID9 | 38 | 223 | BLQ | 12 | BLQ | 10000 | 13037 | 101 | 292 |
| PID10 | 2608 | 93 | BLQ | BLQ | 13 | 7304 | 1444 | 341 | 1236 |

*Table 16: Plasma Cytokines*

| PID | IFN-G | IL-10 | IL-12P40 | IL1A | IL-1B | IL-6 | IL-8 | TNF-A | IP-10 |
|---|---|---|---|---|---|---|---|---|---|
| PID1 | 111 | 5 | BLQ | BLQ | BLQ | 13 | 34 | 31 | 77 |
| PID2 | 17 | 6 | BLQ | BLQ | BLQ | 19 | 25 | 26 | 159 |
| PID3 | 35 | BLQ | BLQ | BLQ | BLQ | 23 | 25 | 35 | BLQ |
| PID4 | 1 | BLQ | BLQ | BLQ | BLQ | 70 | 9 | 10 | 24 |
| PID5 | 317 | 7 | BLQ | BLQ | BLQ | 98 | 156 | 168 | 305 |
| PID6 | 84 | 3 | BLQ | BLQ | BLQ | 22 | 21 | 44 | 153 |
| PID7 | 351 | 18 | BLQ | BLQ | 10 | 12 | 17 | 89 | 52 |
| PID8 | 20 | BLQ | BLQ | BLQ | BLQ | 20 | 15 | 25 | 100 |
| PID9 | 5 | 13 | BLQ | BLQ | BLQ | 88 | 30 | 47 | 86 |
| PID10 | 13 | BLQ | BLQ | BLQ | BLQ | 33 | 3 | 12 | 36 |

*Table 17: Saliva Cytokines*

| PID | IFN-G | IL-10 | IL-12P40 | IL1A | IL-1B | IL-6 | IL-8 | TNF-A | IP-10 |
|---|---|---|---|---|---|---|---|---|---|
| PID1 | 11 | 21 | BLQ | 1040 | 26 | 23 | 127 | 16 | 1061 |
| PID2 | 23 | 46 | 63 | 15644 | 214 | 11 | 401 | 59 | 574 |
| PID3 | 38 | 17 | 58 | 13347 | 1218 | 35 | 1119 | 78 | 3287 |
| PID4 | 2 | BLQ | BLQ | 722 | 17 | BLQ | 58 | 5 | 52 |
| PID5 | 22 | 3 | 11 | 6473 | 146 | 315 | 1393 | 81 | 1184 |
| PID6 | 9 | 26 | BLQ | 1758 | 5 | 1 | 48 | 8 | 133 |
| PID7 | 18 | 87 | 37 | 919 | 25 | 10 | 325 | 13 | 269 |
| PID8 | 22 | 13 | 31 | 3786 | 108 | 9 | 1394 | 12 | 1552 |
| PID9 | 15 | BLQ | 24 | 2999 | 30 | 29 | 486 | 210 | 5945 |
| PID10 | | | | | | | | | |

*Table 18: Cytokine reference ranges and Bioplex200 range and precision*

| Cytokine | Normal plasma levels Median(10th%tile-90th%tile) | Range | | Precision CV% | |
|---|---|---|---|---|---|
| | | LLOQ | ULOQ | Intra | Inter |
| IFN-G | 0.1(0.1-0.3) | 0.03 | 1699 | 9 | 11 |
| IL-10 | 4.8(3.7-8.8) | 0.36 | 8698 | 9 | 11 |
| IL-12P40 | LLOQ(LLOQ-LLOQ) | 0.10 | 2334 | 7 | 7 |
| IL-1A | LLOQ(LLOQ-LLOQ) | 1.68 | 2560 | 4 | 12 |
| IL-1B | LLOQ(LLOQ-LLOQ) | 0.09 | 866 | 9 | 10 |
| IL-6 | 3.4(1.3-10.1) | 0.07 | 1567 | 6 | 6 |
| IL-8 | 4.4(2.4-6.5) | 0.19 | 1818 | 6 | 6 |
| TNF-A | 14.4(11.8-19.3) | 4.79 | 2922 | 6 | 8 |
| IP-10 | 64.4(33.0-103.6) | 0.89 | 541 | 5 | 7 |


1. FDA F. Guidance for industry: bioanalytical method validation. *http://www fda gov/cder/Guidance/4252fnl pdf* 2001.
2. Onlink. Validation Data. 14 April 2023 2023. https://olink.com/content/uploads/2023/02/olink-flex-validation-data.pdf.
